# Comparative effectiveness of intra-arterial thrombolysis vs. mechanical thrombectomy: a literature review

**DOI:** 10.1097/MS9.0000000000003139

**Published:** 2025-04-16

**Authors:** Bisrat Abate Bekele, Olivier Uwishema, Abel Haileyesus Adam, Abdi Degefu Gashu, Charbel Kachouh, Sarah Mshaymesh, Jack Wellington

**Affiliations:** a Department of Research and Education, Oli Health Magazine Organization, Kigali, Rwanda; b School of Medicine, College of Health Sciences, Addis Ababa University, Addis Ababa, Ethiopia; c Department of General Dentistry, Faculty of Dental Medicine, Saint Joseph University, Beirut, Lebanon; d Faculty of Sciences, Haigazian University, Beirut, Lebanon; e Leeds Teaching Hospitals NHS Foundation Trust, Leeds, United Kingdom

**Keywords:** acute ischemic stroke management, clinical outcomes, comparative effectiveness, functional independence, intra-arterial thrombolysis, mechanical thrombectomy, recanalization rates, thrombolytic therapy

## Abstract

**Background:**

Acute ischemic stroke (AIS) remains one of the leading causes of morbidity and mortality worldwide. Time is the single most critical factor in the management of patients presenting with AIS, where re-opening of occluded blood vessels is paramount. Intra-arterial thrombolysis (IAT) and mechanical thrombectomy (MT) are two such therapies employed to reestablish cerebrovascular blood flow in patients with AIS. This review compares both IAT and MT according to their efficacy, safety profiles, recanalization rates, clinical outcomes, and adverse procedural events.

**Methods:**

Study abstraction from electronic search databases comprising PubMed/MEDLINE, ResearchGate, and the National Library of Medicine was used. Screening and selection of relevant articles were comprehensively conducted for this review. Direct comparisons between IAT and MT in terms of variables including recanalization rates, clinical outcomes, and adverse procedural events were warranted for study inclusion. Research determined to exhibit insufficient data or without comparable groups were subsequently excluded.

**Results:**

MT was prompter when executing procedures than IAT, achieving greater rates of recanalization. Both interventions displayed similar results regarding rates of symptomatic intracranial hemorrhage (sICH) and mortality. Despite current available data indicating MT to demonstrate more efficiency as a procedure, further research is needed to examine IAT in light of specific patient demographics, clinical presentation, and circumstances.

**Conclusion:**

This review elucidated that MT often takes less time peri-procedurally, achieving greater success in revascularization compared with that of IAT. Regarding mortality and sICH rates, IAT and MT comparison showed equivocal results. Thus, while making therapeutic decisions, it is important to consider the unique clinical features of each patient as well as the timing of interventions in order to maximize treatment outcomes.

**HIGHLIGHTS**

- Mechanical thrombectomy (MT) demonstrates faster procedural times and higher recanalization rates compared to intra-arterial thrombolysis (IAT) in acute ischemic stroke treatment.
- Both MT and IAT exhibit similar safety profiles regarding symptomatic intracranial hemorrhage (sICH) and mortality rates.
- Further research is needed to explore the efficacy of IAT in specific patient populations and clinical scenarios, despite the overall procedural efficiency of MT.

## Introduction

### Background and the different types of strokes

A stroke, or cerebrovascular accident (CVA), is defined as an abrupt onset of neurological deficit that is attributable to a focal cerebrovascular etiology. The diagnosis of stroke is clinical and is supported by laboratory and radiological findings^[1]^.

There are two types of strokes classified based on pathophysiology:

1. **Ischemic stroke**: a reduction in cerebrovascular blood flow to a brain region, often as a sequalae of an acute thrombosis, embolism, or systemic hypoperfusion.
2. **Hemorrhagic Stroke**: occurs when blood from an artery acutely extravasates into surrounding brain tissue, including intraparenchymal and subarachnoid hemorrhage.

Transient ischemic attacks (TIAs) are short-lived CVAs with symptoms that typically last less than 24 hours. Most TIA symptoms resolve in less than 1 hour. TIAs do not cause permanent damage to the brain but often herald a risk for future strokes. If signs of infarction are detected by means of radiological testing, the condition is classified as a stroke, irrespective of how long the symptoms last. If neuroimaging post-TIA reveals no significant dynamic changes, a consequent exclusion of TIA is not recommended; instead, the clinical presentation is considered diagnostic.

### Prevalence and impact of ischemic stroke

Ischemic stroke accounts for 80% of all strokes, with the remaining 20% being hemorrhagic in nature. Such CVAs rank among the leading causes of morbidity and mortality globally, affecting both high-income and low-income nations^[2,3]^. More than two-thirds of new cases occur due to ischemic-type stroke alone^[3]^. It has become a major global health concern due to rising incidence rates, particularly in Asian nations like that of the People’s Republic of China, which has reported the highest number of cases to date^[4]^. Nevertheless, its prevalence remains higher in developed regions compared to developing ones, where other medical conditions, like that of communicable infectious disease, may have a greater impact on public health. The overall incidence rate is estimated at 2–2.5 per thousand population internationally accompanied by a 5 year recurrence rate ranging between 15% and 40%^[5]^. It poses significant burden in terms of disability-adjusted life years (DALYs) lost particularly among individuals aged 65–69 years old^[4]^.

Acute ischemic stroke (AIS) caused by large vessel occlusion (LVO) exhibits worse clinical outcomes as it leads to more pronounced areas of cerebral infarction, more profound symptoms, and poorer long-term prognoses^[6]^. A study performed in the United Kingdom (UK) demonstrated an estimated 30% of individuals within 1 year to succumb to mortality as new cases of AIS while another 35% became permanently disabled and dependents^[7]^. Though intravenous thrombolysis and mechanical thrombectomy (MT) have shown promising results in some studies, these procedures are not readily available globally^[3]^. Given these facts, preventive measures should be put in place to combat growing burden posed by this condition.

### Background on stroke treatment

Rapid initiation and delivery of appropriate therapy for AIS is paramount since it greatly affects patient outcomes. The golden period for stroke management usually falls within 4.5 hours post-symptom onset when considering intravenous thrombolysis. This extends up to 6 hours if MT can be performed but may be prolonged to 24 hours based on advanced imaging findings in selected patients, i.e., through means of diffusion-weighted magnetic resonance imaging (DW-MRI)^[8,9]^. Delaying treatment is associated with increased morbidity and mortality^[8]^.

Intravenous thrombolysis is a current treatment paradigm that may dissolve the thrombosis/embolism when administered within 4.5 hours following symptom onset. MT, which involves physical removal of a thrombus or embolus, is recommended to be performed within 6 hours; however, it is extendable to 24 hours if appropriate imaging is available (i.e., DW-MRI)^[8^-^10]^. Functional outcomes are significantly improved with MT compared to thrombolytic therapy alone, as it reduces mortality rates and improves functional outcomes^[9,11]^. These two methods are often combined together in order to restore cerebrovascular blood flow^[12]^.

### Intra-arterial thrombolysis and mechanical thrombectomy: an overview

Intra-arterial thrombolysis (IAT) is a process in which thrombolytic substances like that of urokinase or recombinant tissue plasminogen activator (rtPA) are directly delivered via a catheter into the clot site, with the aim of dissolving thrombi or emboli and restoring cerebrovascular blood flow^[13,14]^. On the contrary, MT comprises the application of instrumentation including stent retrievers or aspiration catheters to physically remove the arterial blood clot^[15,16]^.

The primary objective of IAT is chemical lysis of clots, leading to improvement in microcirculatory reperfusion^[14]^. Rapid complete recanalization with short recanalization time is aimed at by MT through mechanical extraction of clots, which may result into higher rates for achieving a more complete recanalization faster^[15,16]^. Both treatments seek to reestablish blood flow to prevent further brain damage and enhance clinical outcomes.

### Purpose and scope of the literature review

The primary objective of this review was to evaluate the efficacy of IAT and MT in the management of AIS in terms of clinical outcome metrics, including mortality, symptomatic intracranial hemorrhage (sICH), and recanalization rates. Among the many factors pertaining to IAT and MT that will be examined in this study are procedural results, recanalization rates, and clinical efficacy. Certain studies claim that MT exhibits prompter procedural times in addition to higher degrees of recanalization than IAT^[15,17]^. Nevertheless, there was no difference in the clinical results, such as sICH and mortality rates, between these two regimens^[11]^. Additionally, this study aimed to acknowledge the shortcomings and diversity in methods employed by different scholars over the course of their research. Moreover, it will recognize any potential biases that could have been precipitated by variations in patient demographics or treatment regimens.^[17,18]^.

## Materials and methods

A review was conducted using a study abstraction approach from electronic search databases, including PubMed/MEDLINE, ResearchGate, and the National Library of Medicine. Comprehensive screening and selection of relevant articles were performed to ensure the inclusion of studies directly comparing IAT and MT for the management of AIS. Key variables for comparison included recanalization rates, clinical outcomes, and adverse procedural events. Studies with insufficient data or lacking comparable groups were excluded.

The search strategy utilized a combination of strategically chosen terms, including “acute ischemic stroke management,” “mechanical thrombectomy,” “intra-arterial thrombolysis,” and “thrombolytic therapy,” along with relevant keywords such as “clinical outcomes,” “comparative effectiveness,” “functional independence,” and “recanalization rates.” This approach enabled the inclusion of all pertinent studies on the topic. Only English-language publications were considered, and a meticulous analysis of the existing literature was conducted to assess the comparative effectiveness of IAT and MT in managing AIS.

## Overview of intra-arterial thrombolysis

Thrombolysis is the process of established clot diminution to recanalize an artery. Both intravenous thrombolysis and IAT have been employed to manage AIS. The more recent introduction of IAT involves the endovascular insertion of a catheter to deliver a thrombolytic agent at or near the site of the clot. This allows for a more targeted treatment and decreased systemic toxicity. Compared to intravenous thrombolytic administration, IAT also allows a longer window period at which it can be performed.

The discovery of fibrinolytic activators in the 1930s led to advances in AIS management. However, initial fibrinolytic treatment of AIS in the 1950s faced hurdles due to the absence of computed tomography (CT) scans (until the 1970s) and nonspecific agents like that of urokinase and streptokinase, resulting in high ICH rates^[19]^. The introduction of rt-PA in 1983 marked a significant milestone^[1]^. The application of rt-PA demonstrated efficacy in AIS treatment in 1995 when administered within 3 hours of symptom onset. Despite revolutionizing AIS management, rt-PA has limitations, such as a narrow therapeutic window and bleeding risk. These prompted exploration of other treatment options like IAT, which showed promise in the ProACT I & II^[20]^ trials using pro-urokinase, despite an increased ICH risk. Subsequent smaller trials like that of MELT (Middle Cerebral Artery Embolism Local Fibrinolytic Intervention)^[21]^ and IMS (Interventional Management of Stroke)^[22,23]^ further evaluated IAT with or without intravenous thrombolysis, but IAT remains FDA-unapproved for AIS management. Table 1 represents the key findings of IAT.

**Table 1:** Key trials and findings in IAT

Trial	Thrombolytic Agent	Main Outcomes	ICH Risk	References
ProACT I & II	Pro-urokinase	Increased recanalization, not FDA approved, higher ICH risk	High	^[20]^
MELT	Urokinase	Evaluated efficacy, discontinued due to rt-PA approval	Observed to be higher but did not reach statistical significance	^[21]^
IMS	Various (with/without IV rt-PA)	Emphasizes the use of combined IV/IAT rather than IAT alone	Higher (similar to previous studies)	^[22,23]^

Since IAT is not FDA-approved as of yet, there remains a great deal of variability between experts in the field on the indications of IAT. In general, IAT may be offered to patients with CVAs who do not meet criteria for intravenous rt-PA administration because of time window constraints or other clinical co-factors^[24]^. Patients must also have clinical deficits that are severe enough to warrant intervention, most likely due to LVO or high clot burden (>8 mm)^[25]^.

The only thrombolytic proven in large clinical trials, i.e. ProACT trials^[20]^, is pro-urokinase. The MELT trial^[21]^ assessing efficacy of IAT evaluated urokinase application but was prematurely discontinued attributable to the approval of intravenous rt-PA in AIS management. There are also other smaller studies evaluating efficacy of different thrombolytics in AIS management, including post-operative ischemic stroke^[7]^.

The fact that IAT requires more time than intravenous thrombolysis is one of its drawbacks. Another pertains to the invasiveness of such a procedure, where a greater risk of allergic reaction/anaphylaxis, acute renal failure, transient hypotension, and hematomas at the access site. The procedural costs could also be a significant barrier, but they might be balanced by the decrease in expenses related to long-term morbidity^[26]^.

## Overview of mechanical thrombectomy

MT is the physical removal of an obstructing intravascular clot to facilitate cerebrovascular blood flow to resume. It is performed via catheterization of a peripheral artery, commonly the femoral artery. The catheter is then advanced to the site of the intracranial artery occlusion. A stent retriever inserted into the catheter is then used to retract and remove the clot. Catheter aspiration devices are an alternative to stent retrievers and may aspirate the thrombosis/embolism as the first approach. If unsuccessful, stent retrievers may be employed.

The small-time window period (<3 hours) and low recanalization rates with intravenous thrombolytics in the treatment of AIS inspired the evolution of endovascular stroke management.^[27]^ Initial trials, like that of the ProACT^[20,28]^ trial, employed IAT to tackle these shortcomings. Despite showing an increase in recanalization rates and window periods, the FDA did not approve these due to their small sample sizes and marginal statistical significance.

The development of MT devices led to the advancement of endovascular treatment. The MERCI trial^[29,30]^, which used the MERCI device for MT, showed much higher recanalization rates and was approved by the FDA. However, the non-randomized trial design and higher mortality rates had fueled some controversy at the time.

The development of stent retrievers and other second-generation devices marked a significant advance by achieving recanalization rates of up to 90%. These devices were validated through a number of trials including, the MR CLEAN trial^[31]^, which announced its positive results for anterior circulation AIS in late 2014 when intra-arterial intervention (IAT, MT, or both) was performed within 6 hours. Other trials, such as ESCAPE^[32]^ and SWIFT PRIME^[33]^, were discontinued early because of positive interim efficacy in the MT treatment group. The HERMES meta-analysis^[34]^, which assessed 1287 patients from multiple trials, including those mentioned above, showed significantly increased rates of functional independence and reduced disability with no significant difference in rates of sICH. The DAWN^[9]^ and DEFUSE 3^[35]^ trials also demonstrated positive results when MT was performed within 6–24-hour window periods.

Despite the extensive evidence supporting the application of MT in anterior circulation AIS, trials showing its benefit in posterior circulation strokes are sparse. The ATTENTION^[36]^ and BAOCHE^[37]^ trials both demonstrated clinical benefits, but both were performed in a population with a predominance of Chinese ancestry. Other trials were limited by either methodologic issues or a lack of statistical significance. Table 2 shows key findings of trials of mechanical thrombectomy.

**Table 2:** Key trials and findings in mechanical thrombectomy

Study	Included patients	Outcomes	Reference
MERCI I	Patients with NIHSS ≥ 10 and presenting between 3 to 8 hrs. after symptoms	Increased recanalization rates (some patients also received IAT), improved functional outcome	^[29]^
MR CLEAN	Patients with proximal anterior intracranial artery occlusion who presented within 6 hours of symptoms	Increased recanalization rates (although not as high as previous studies), improved functional outcome, 9% had embolization to another territory	^[31]^
DEFUSE 3	Patients who presented between 6 and 16 hours of stroke onset and have internal carotid or middle cerebral artery occlusions	Aimed to establish objectively which patients will be benefit from late revascularization	^[35]^
ATTENTION	Patients with basilar artery stroke who presented with basilar artery stroke	Better recanalization rates and functional status; reduced mortality rate; but, 5% had sICH and 1 patient died of arterial perforation	^[36]^
BAOCHE	Patients with basilar-artery stroke who presented between 6 and 24 hours after symptom onset	Better functional status but increased rate of sICH (6% vs. 1%)	^[37]^

Careful selection of patients eligible for MT is important to avoid unnecessary procedures. Although individualized decision-making is necessary, patients that would benefit from MT include patients whose strokes have been caused by a LVO in the anterior circulation and have been at baseline status within the past 24 hours. The neurologic deficits should also be persistent and potentially disabling, i.e., National Institutes of Health Stroke Scale (NIHSS) of ≥6. Imaging (i.e., CT without contrast or DW-MRI) should rule out hemorrhage and show an Alberta Stroke Program Early CT score^[38]^ (ASPECTS) ≥ 3. Patients with NIHSS <6, large infarct core (ASPECTS <3 or core infarct >70 mL), poor baseline functional status (modified Rankin Scale (mRS) >1), and distal occlusion are generally excluded as any benefit is outweighed by adverse events^[39]^.

## Comparative effectiveness of IAT and MT

CVA, commonly known as acute stroke, is a major medical emergency endangering the patient’s vital prognosis and is characterized by the sudden onset of neurological deterioration caused by intrinsic cerebrovascular pathologies^[40,41]^. IAT and MT were suggested therapies by recent evidence. These two therapeutic approaches may provide benefits for ischemic stroke patients^[42,43]^. Alteplase and various thrombolytic agents were developed to help in reducing ischemic stroke severity^[44]^. Early administration of intravenous alteplase can enhance neurological improvement by increasing the odds of early neurological improvement (ENI) in patients with moderate stroke severity^[45]^. However, ENI is rarely observed without thrombectomy in patients with LVO^[46]^. Clinical outcomes such as recanalization rates, functional outcomes, and mortality rates remain essential measurable changes in assessing the efficacy of these different therapeutic modalities.

**Figure 1.**
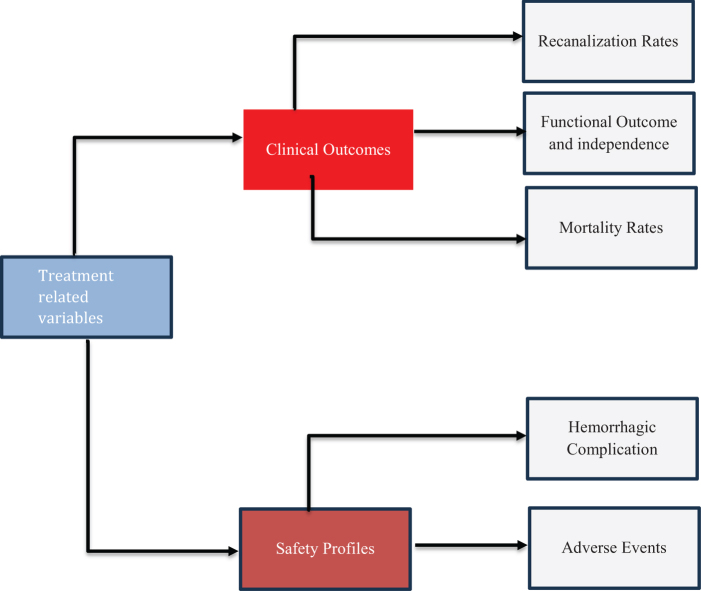
Treatment related variables.

The mRS quantifies post-stoke disability in everyday practice^[47]^. This scale grades and sorts out a patient’s disability, ranging from 0 to 6, representing no symptoms and state of death, respectively^[48,49]^. When MT is unsuccessful for AIS patients, IAT may be considered as a safe rescue option therapy^[50]^. IAT has also been used along with MT in cases where MT did not achieve sufficient restoration of cerebrovascular blood flow. Figure 1 depicts the treatment-related variables that must be considered when deciding between IAT and MT.

As the incidence of LVO increases worldwide, evaluating the efficacy of IAT and MT becomes increasingly important. Incomplete recanalization postprocedure may occur due to technical difficulties related to clot retrieval^[51]^. Thrombus fragmentation, distal territory inaccessibility, and fibrin-rich thrombus composition are currently the most significant^[52]^. Recanalization rates vary between the two therapies.

The ProACT II trial^[20]^ studied a randomized population of 180 subjects with middle cerebral artery occlusion to receive either intra-arterial pro-urokinase plus heparin (study group: *n* = 121) or heparin only (control group: *n* = 59). The primary outcome, proportion of patients with an mRS score of 2 or less, was 40% in the pro-urokinase group compared to the 25% in the control group (*P* = 0.04). The recanalization rate was significantly higher in the study group, but so was ICH with clinical deterioration within 24 hours.

The HERMES trial^[34]^ combined five trials on MT versus standard non-endovascular management on patients with AIS secondary to proximal anterior LVO. It analyzed 1287 patients, with 634 patients having endovascular thrombectomy and 653 assigned to the control group. The study showed that patients assigned to MT had better functional outcomes as measured by mRS scores at 90 days, NIHSS scores at 24 hours, and change in NIHSS scores from baseline to 24 hours. The incidence of sICH, 4.4% in the MT group vs. 4.3% in the control group, was also not significantly increased. Mortality also did not differ between the groups significantly. Table 3 represents the comparison between IAT and MT.

**Table 3:** Comparison of IAT and MT

	Intra-arterial thrombolysis	Mechanical thrombectomy	References
Recanalization rates	66%	83%*–*94%	^[20,33,53]^
mRS score 0–2 at 90 days	40%	46%	^[20,34]^
sICH	10%	4.4%	^[20,34]^
Mortality rate	25%	15.3%	^[20,34]^
Adverse events	Bleeding, allergic reactions, angioedema, anaphylactic reaction, arrhythmias	Device related complications, vessel perforation	^[54-56]^

As improving quality of life remains essential among all medical fields, the importance of functional outcomes and independence cannot be overstated^[57]^. A patient’s ability to perform daily tasks post-recovery is quite essential. The Post-Acute Care Cerebrovascular Diseases (PAC-CVD) provides the opportunity to investigate this issue^[52,58,59]^. Age, balance, leg strength, and cognition can dictate poststroke walking performance and ability for each patient^[60]^.

Safety profiles for all treatments are essential, as they dictate possible complications. Hemorrhagic complications and adverse events may be associated with IAT and MT. Bleeding, hypertension, allergic reactions, angioedema, anaphylactic shock, and reperfusion arrythmias are encountered adverse events following IAT. Bleeding remains the most frequent complication happening at the puncture site or anywhere inside the body^[61]^. Adverse events for MT are mostly device-related and vessel perforation. Despite several advancements in MT devices and maneuvers resulting in high reperfusion rates in patients suffering from LVO, ICH remains the most occurring complication, with a reported incidence as high as 40% in some studies^[54]^. With an incidence ranging from 1 to 4%, stent retriever (SR) detachment may occur when treatment for anterior and posterior circulation is being performed^[55,56,62]^.

An article^[63]^ directly compared the efficacy of MT and IAT in patients with minor strokes (NIHSS <5) secondary to LVO, as confirmed by digital subtraction angiography. This study found IAT to be superior to MT in various areas. A total of 120 patients received either IAT (63 patients) or MT (57 patients) as the primary treatment strategy. When compared to patients who had undergone MT, those who had received IAT had a higher chance of functional recovery as measured by the 90-day mRS scores and a lower chance of 90-day mortality. They also had much shorter puncture-to-recanalization times. The incidence of any ICH within 48 hours was increased in patients who underwent MT. Although this study showed clear superiority of IAT, its generalizability is questionable as it only used patients with mild functional decline initially. Table 4 shows the comparison of IAT and MT in patients with mild acute ischemic stroke.

**Table 4:** Comparison of IAT and MT in patients with mild AIS, i.e. NIHSS <5

	Intra-arterial thrombolysis	Mechanical thrombectomy
Successful recanalization rates	90.5%	89.5%
Puncture to recanalization time	Shorter	Longer
Risk of ICH	Much lower	Higher
Mortality rate at 90 days	Much lower	Higher

Cost-effectiveness and long-term financial impact are indeed important as well. Cost-effective analysis (CEA) has become a crucial component in new health technology valuation as it aids decision-making by assessing if a new treatment approach is worth the additional cost^[64]^. The long-term financial impact of stroke management remains an essential burden for people. Currently, an alarming rate of younger patient age groups is being recognized^[65]^. The financial impact of stroke is high due to long hospital care and long-term rehabilitation management^[66]^. MT can be seen as independently cost-effective among various health systems^[67]^. Patients suffering from LVO could be treated with MT, as it is more economically sustainable when compared or added to medical care and management^[68,69]^. As for patient outcomes, when compared to MT, IAT may improve 90-day clinical patient outcomes with reduced ICH and mortality rates in LVO patients, but this is an area that requires further research^[63]^.

## Challenges, recent advances, and innovations

### Challenges

The effectiveness of IAT and MT faces some issues:

- Rate of Recanalization and Time Sensitivity: MT has shown high rates of recanalization but does not achieve substantial reperfusion (mTICI 2b-3) in about 30% of cases or when it is performed too late. The time taken for reperfusion is significant as it directly affects patient outcomes^[70]^. If total ischemic time exceeds 7.3 hours, MT efficacy decreases drastically.
- Thrombus Characteristics: It is harder to retrieve fibrin-rich thrombi, which possess much friction and adhesion. Also, if the length of a clot increases, then removal becomes more complicated due to increased friction as well as adhesion.

Problems with Vascular Access: When issues prevent arterial access, such as peripheral vascular disease or an anatomically anomalous aortic arch, difficulties may arise peri procedurally. Direct carotid puncture may be required in some circumstances, although there are risks associated with it, including the possibility of cranial nerve damage and respiratory compromise. Additional challenges posed include the possibility of sICH from IAT, distal embolization with procedural issues such as arterial dissection, and hemorrhage^[71]^. This increases the risk and limits the application of sophisticated imaging for patient selection. To overcome these challenges, it is necessary to continue researching and refining techniques so as to improve outcomes while minimizing risks to patients treated^[72,73]^.

### Advances in thrombolytic agents

In recent times, there have been improvements made on AIS treatment by concentrating on enhancing thrombolytic agents together with MT devices^[72]^. Although the only FDA-approved drug for managing this condition is intravenous rtPA, intra-arterial interventions have proven beneficial among patients who cannot receive rtPA^[74,75]^. Some new studies are being conducted to assess whether Tenecteplase may be used as a thrombolytic agent.

### Innovations in thrombectomy devices and techniques

From the first-generation Merci retrieval systems to second-generation Penumbra aspiration systems and third-generation stent retrievers, MT has evolved over time^[74,76]^. These advancements have greatly improved outcomes for individuals suffering from AIS. It was observed that reperfusion rates may be enhanced by second-generation devices like those of the Solitaire and Trevo stent retrievers^[9]^. Additionally, clot retrieval has been further optimized through techniques such as the “pinning technique” or using large-bore aspiration catheters for this purpose^[12]^. Combining rtPA or glycoprotein IIb/IIIa inhibitors with MT via the intra-arterial route may improve functional outcome while reducing fatality rates. In addition, when compared with other methods of recanalization, balloon guide catheters were found to achieve faster recanalization, leading to better clinical outcomes^[8]^.

### Combined approaches and adjunctive therapies

Good patient outcomes have been shown by combined approaches together with adjunctive therapies during MT treatment for AIS. When such methods are used simultaneously, they may enhance functional outcome without increasing risk for sICH, which may occur during intra-arterial administration of IATs like urokinase or glycoprotein IIb/IIIa inhibitors^[77]^. “Pinning technique,” which combines local aspiration with stent retrievers, achieves greater clot extraction strength while minimizing distal embolization during this process. Moreover, when balloon guide catheters are used during stent retriever thrombectomy, there is prompter recanalization rates, facilitating better clinical outcomes^[8]^.

### Emerging technologies and what may come next

Among other variables, larger and more flexible aspiration catheters, alongside improved stent retrievers with radial force adaptability, are being developed to improve the pace of revascularizing occluded cerebrovascular arteries and optimize patient clinical status post-stroke^[8]^. There have been refinements in methods used to improve clot retrieval, like the “pinning technique” or using intermediate catheters for local aspiration^[12]^. Hence, future developments aim at device innovation that may interact better with thrombi or emboli, where further research regarding IAT is warranted, which could be employed for managing small clots occluding distal cerebrovascular arteries, thus improving tissue perfusion fed by these arteries^[77]^. Additionally, advanced imaging techniques are being investigated to better select patients for MT^[9]^.

## Conclusion

### Synthesis of discoveries

A scientific investigation compared the efficacy of IAT and MT in AIS management. Key findings suggest MT generally exhibits higher rates of revascularization of cerebrovascular blood vessels alongside shorter procedure times when compared to IAT. The incidence of good functional outcomes and adverse procedural events varied in different studies. However, MT seems to possess better patient outcomes and is FDA-approved. The scarcity of large trials studying the efficacy of IAT and the borderline statistical significance observed in the ProACT II trial have hindered FDA approval for IAT.

### Strengths and weaknesses of the reviewed studies

The comprehensive evaluation of clinical outcomes, recanalization rates, and adverse events by the reviewed studies is one strength, as it provides a wide-ranging view on how well IAT compares with MT in managing AIS.^[19,20]^ However, there are also certain restrictions: large-scale randomized controlled trials directly comparing these two approaches were lacking, limiting generalizability. Alternatively, varying study designs may have skewed results due to differences among patients being treated or procedures used during their conduct may have been flawed. Some studies were not blinded, study investigators may well have concluded what treatment each patient had received when assessing their outcome measures, such as functional independence scales like mRS, potentially creating a placebo effect^[21,22]^.

### Final thoughts on the comparative effectiveness

Despite MT typically producing greater mechanical efficiency and therapeutic results than that of IAT, there are still circumstances in which one therapy may be more effective than the other. Consequently, the choice between procuring these two approaches should be based on personal factors like age, degree of severity, and time period, as well as availability of necessary resources and experienced staff at different institutions^[41]^. Both treatment methods have their own advantages and provide their specific advantages in the management of AIS. Depending on particular patient conditions, utilizing them in combination may be superior to a single-modal approach^[49]^. The findings in this review support the use of MT as the mainstay management for individuals with anterior circulation LVO while also recognizing the place of IAT in specific scenarios. Implementing these recommendations may result in better patient outcomes by lowering the rates of long-term disabilities and improving the overall management of AIS^[47]^. Moreover, it may increase functional recovery and improve quality of life for post-stroke survivors^[78]^.

### Recommendations for future research

Further study and research with future randomized controlled trials that directly compare both IAT and MT is warranted to provide more definitive evidence regarding their comparative efficacy and safety profile in the management of AIS. Additionally, data pertaining to long-term clinical outcomes following IAT and MT is necessitated by considering factors comprising cost-effectiveness and improving patient-centered outcome metrics such as quality-adjusted life years and DALYs saved alongside health policymaking processes in various settings^[44]^. An additional domain of inquiry pertains to the potential hybrid application of these two therapeutic modalities to yield the best outcomes for ischemic stroke patients^[48]^.

## Data Availability

Not applicable.
